# Hydrogel-Based Bioelectronics and Their Applications in Health Monitoring

**DOI:** 10.3390/bios13070696

**Published:** 2023-06-30

**Authors:** Jiangbo Hua, Mengrui Su, Xidi Sun, Jiean Li, Yuqiong Sun, Hao Qiu, Yi Shi, Lijia Pan

**Affiliations:** Collaborative Innovation Center of Advanced Microstructures, School of Electronic Science and Engineering, Nanjing University, Nanjing 210093, China

**Keywords:** hydrogel, bioelectronic, health monitoring

## Abstract

Flexible bioelectronics exhibit promising potential for health monitoring, owing to their soft and stretchable nature. However, the simultaneous improvement of mechanical properties, biocompatibility, and signal-to-noise ratio of these devices for health monitoring poses a significant challenge. Hydrogels, with their loose three-dimensional network structure that encapsulates massive amounts of water, are a potential solution. Through the incorporation of polymers or conductive fillers into the hydrogel and special preparation methods, hydrogels can achieve a unification of excellent properties such as mechanical properties, self-healing, adhesion, and biocompatibility, making them a hot material for health monitoring bioelectronics. Currently, hydrogel-based bioelectronics can be used to fabricate flexible bioelectronics for motion, bioelectric, and biomolecular acquisition for human health monitoring and further clinical applications. This review focuses on materials, devices, and applications for hydrogel-based bioelectronics. The main material properties and research advances of hydrogels for health monitoring bioelectronics are summarized firstly. Then, we provide a focused discussion on hydrogel-based bioelectronics for health monitoring, which are classified as skin-attachable, implantable, or semi-implantable depending on the depth of penetration and the location of the device. Finally, future challenges and opportunities of hydrogel-based bioelectronics for health monitoring are envisioned.

## 1. Introduction

With the increase in public health awareness, people’s concern about their health is no longer restricted to special scenarios such as hospitalization. Health monitoring in daily life is also receiving more and more attention [1,2,3]. Despite the fact that conventional health monitoring devices can offer real-time monitoring of human health status, they are mostly rigid and cumbersome to operate [4]. In recent years, flexible bioelectronics have emerged as a promising alternative for health monitoring due to their compact size, uncomplicated structure, and comfortable wearability [5,6,7]. They are now available for basic human physiological signal monitoring [8,9,10] and disease screening [11]. The commonly used materials for flexible bioelectronics encompass low-dimensional nanomaterials and conductive polymers, which are largely biocompatible and possess excellent mechanical properties [12,13].

Hydrogel is a water-swollen and cross-linked polymer network. Apart from water as the dispersion medium, hydrogels mainly consist of polymer chains and other dispersed phases [14,15]. Due to the three-dimensional network structure of hydrogel, hydrogels feature a water-based polymer system that is compatible with biological tissues, which imparts good biocompatibility and low Young’s modulus to hydrogel [16]. These characteristics, which are similar to those of human tissue, make hydrogel one of the preferred materials for bioelectronics [17,18]. Conventional hydrogels are mostly networked with insulating hydrophilic polymers that are unable to respond to external stimuli, which makes them unsuitable for health monitoring. By adding conductive polymers, conductive fillers, or free ions to hydrogel, the conductivity of the hydrogel can be modulated, resulting in a conductive hydrogel suitable for health monitoring bioelectronics [19,20]. Conductive hydrogels have also recently been utilized as peripheral neural interfacing devices to deliver electricity with peripheral nerves [21]. Depending on the conductive mechanism, hydrogel can be classified as electronically conductive or ionically conductive hydrogel.

Apart from conductivity, hydrogels can achieve excellent mechanical properties, self-healing, adhesion, and biocompatibility through adjusting the composition and other methods. These properties make hydrogel-based bioelectronics have good stability and avoid an immune rejection reaction with the human body. The self-healing ability of hydrogels has been dramatically improved by the inspiration of natural organisms [22]. To make health monitoring more accurate, the sensing interface based on the hydrogel needs to adhere to the skin. To strengthen the property of adhesion, chemical bonds, topological structures, and their synergy are widely taken into consideration [23]. Meanwhile, with the addition of suitable particles, the strength of chemical bonds is increased, which enables hydrogels to have both high mechanical strength and strong adhesion to interfaces while maintaining good biocompatibility, broadening the application scope of hydrogel-based bioelectronics [24]. Therefore, hydrogel-based bioelectronics have been widely used in health monitoring fields such as biosensors, flexible electrodes, and ultrasound detection [25,26,27,28,29].

**Figure 1:**
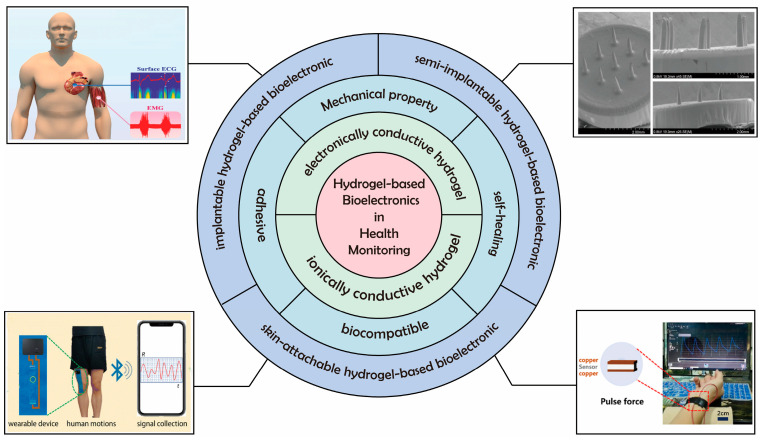
Hydrogels with different properties for health monitoring. Reprint with permission from Ref. [30]. Copyright 2022 John Wiley & Sons; reprint with permission from Ref. [31]. Copyright 2019 Elsevier; reprint with permission from Ref. [32]. Copyright 2023 The Royal Society of Chemistry; reprint with permission from Ref. [33]. Copyright 2019 American Chemical Society.

In this review, we comprehensively elaborate on the progress of research on hydrogel-based bioelectronics in terms of the basic properties of hydrogels, material synthesis, and health monitoring applications (Figure 1). Firstly, we focus on the mechanisms and methods to improve the important properties of hydrogels such as conductivity, mechanical properties, self-healing, adhesion, and biocompatibility. Then, we describe the application of hydrogel-based bioelectronics for health monitoring. By integrating hydrogels into diverse forms of devices, hydrogel-based bioelectronics can be used in a skin-attachable, semi-implantable, or implantable form for physical and chemical vital signs signal monitoring. Finally, we address the current challenges and prospects for the future development of hydrogel-based bioelectronics. 

## 2. Properties of Hydrogels

Health monitoring requires devices to convert the information obtained into a measurable output. Currently, the most widely used method is to enable hydrogels to acquire conductivity and give them the ability to output electrical signals. In addition, because hydrogel has mechanical strength, self-healing, adhesion, biocompatibility, and other exceptional properties that make it superior to other materials, it has become a promising material for wearable devices [34,35,36]. In this section, we discuss the five main properties of hydrogels and the current status of their development, focusing on research advances in optimization methods for the properties of hydrogel. 

### 2.1. Conductivity

Hydrogels consist of a three-dimensional network structure composed of hydrophilic materials, and the cross-linked network can store large amounts of water, ensuring the softness of hydrogels for the preparation of flexible sensors. However, most hydrogels are based on an insulating polymer network matrix, which makes them less conductive and prevents efficient transmission of electrical signals in hydrogel-based bioelectronics.

Improving the electrical conductivity of hydrogels is usually achieved in two ways: by introducing a conductive polymer into the hydrogel matrix or incorporating a conductive filler such as a conductive salt into the conductive hydrogel. Depending on the source of conductivity, hydrogels can be classified into electronically conductive hydrogels or ionically conductive hydrogels.

#### 2.1.1. Electronically Conductive Hydrogels

Electronically conductive hydrogels rely on electron transfer as carriers to conduct electricity and possess excellent environmental stability and conductivity. Typically, electronically conductive hydrogels are prepared by doping conductive polymers (CPs) into the hydrogel matrix to form a conductive network or by adding metal nanoparticles, carbon nanotubes, etc., as conductive fillers to provide free electrons to the hydrogel.

**Figure 2:**
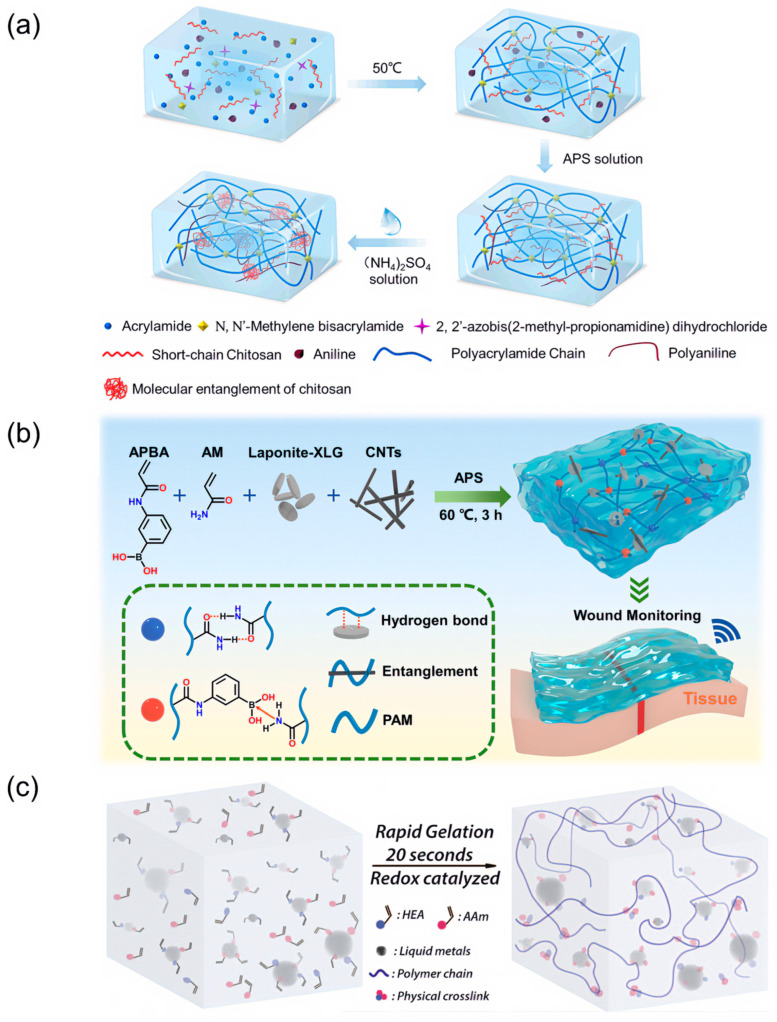
Electronically conductive hydrogel. (**a**) Schematic diagram of the synthesis of dual-network hydrogels. Reprint with permission from Ref. [42]. Copyright 2021 American Chemical Society. (**b**) Schematic diagram of the fabrication of hydrogels via a facile one-step copolymerization of 3-acrylamidophenylboronic acid and acrylamide in the presence of LAPONITE^®^ XLG nanosheets dispersed carbon nanotubes. Reprint with permission from Ref. [43]. Copyright 2023 The Royal Society of Chemistry. (**c**) Schematic diagram of the synthesis of liquid metal hydrogels. Reprint with permission from Ref. [46]. Copyright 2019 The Royal Society of Chemistry.

CPs, commonly including polyaniline (PANI), polypyrrole (PPy), and poly(3,4-ethylenedioxythiophene) (PEDOT), often have a π conjugated structure that allows electrons to move along the polymer backbone to form an electric current [37,38,39,40]. CPs can be co-polymerized with the hydrogel matrix or polymerized in situ in an already formed hydrogel network to form a double network hydrogel, providing a conductive channel for the hydrogel [41]. Cong et al. [42] prepared electronically conductive hydrogels, using chitosan and polyacrylamide (PAAm) doped with PANI, with a double network structure formed by hydrogen bonding between the networks. The dual-network hydrogel maintained good electrical conductivity under extreme environments and could be applied to multifunctional detection of human activities (Figure 2a).

Electronically conductive hydrogels can also be prepared by introducing high conductivity materials. Carbon-based electronic materials such as carbon nanotubes (CNTs) and graphene are often used as conductive fillers in hydrogels due to their superior electrical conductivity. However, the natural hydrophobicity of carbon-based materials can lead to their tendency to aggregate in hydrogel networks, preventing them from forming a homogeneous and stable conductive system. Shen et al. [43] introduced B-N coordination by adding acrylamide and 3-acrylamidophenylboronic acid, and used LAPONITE^®^ XLG (XLG) nanosheets to induce uniform dispersion of CNTs, ensuring the reliability of the electronically conductive hydrogel output. The B-N coordination in the polymers also gives the hydrogels (P(AM-APBA)XLG/CNTs) excellent mechanical properties (Figure 2b). In addition to carbon-based materials, metallic materials are also more commonly used, usually in the form of nanoparticles or nanowires, as conductive fillers for hydrogels [44,45]. Liquid metals with flexibility and high conductivity are currently available as fillers for hydrogels, such as the liquid metal gallium with a melting point of 29.1 °C (Figure 2c) [46]. Other low-dimensional nanomaterials such as MXene can also be added to hydrogels to enhance the electronic conductivity of the hydrogel [47,48].

#### 2.1.2. Ionically Conductive Hydrogels

In inorganic materials, electrical currents are often conducted by the transport of electrons. However, the directional movement of charged ions also enables the transmission of electrical currents, which is crucial to the transmission of physiological signals in living organisms [49]. Hydrogels possess a loose porous structure, which creates the conditions for the preparation of ionically conductive hydrogels [50]. By doping with different fillers, ionic conductivity similar to electrical signal transmission in living organisms can be achieved in hydrogels [51,52]. The ionic conductivity of hydrogels can usually be improved by incorporating inorganic salts and ionic liquids to provide free ions that can move in a directional manner, making them ideal for the preparation of flexible bioelectronics [53,54,55].

**Figure 3:**
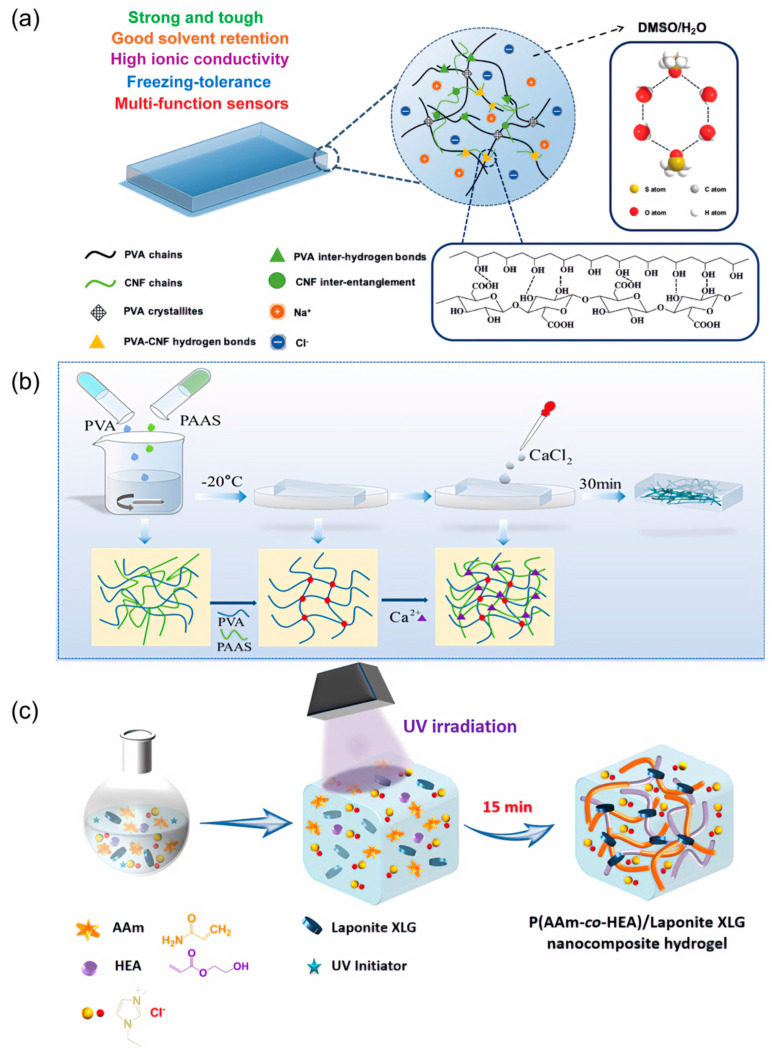
Ionically conductive hydrogel. (**a**) Schematic diagram of polyvinyl alcohol (PVA) and cellulose nanofibers (CNF) organohydrogel. Reprint with permission from Ref. [56]. Copyright 2020 John Wiley & Sons. (**b**) Schematic diagram of the synthesis of PVA, polyacrylate sodium (PAAS), and CaCl_2_ hydrogel. Reprint with permission from Ref. [57]. Copyright 2022 American Chemical Society. (**c**) Schematic diagram of the synthesis of poly(acrylamide-co-2-hydroxyethyl acrylate)/XLG nanocomposite ionogel. Reprint with permission from Ref. [65]. Copyright 2021 American Chemical Society.

Immersion of the hydrogel in a conductive salt solution is the most common method for preparing ionically conductive hydrogels. Ye et al. [56] prepared high conductivity ionic hydrogels by the sol-gel synthesis method using polyvinyl alcohol (PVA) and cellulose nanofibers (CNF) with conductivity up to 3.2 S/m at room temperature (Figure 3a). Although the immersion method has been extensively studied, there is still a need to overcome the problem of low conductivity of ion-conducting itself to expand the application of ionically conductive hydrogels for flexible sensors and energy storage devices. To overcome the problems of uneven ion distribution or insufficient cross-linking brought by the conventional immersion method, Liu et al. [57] proposed to prepare highly conductive ionic hydrogels by freezing cross-linking (Figure 3b). The cryo-cross-linked PVA is used as the matrix of the hydrogel, and the calcium ions in polyacrylate sodium (PAAS) and CaCl_2_ are coupled to form a cluster network structure in PVA to make the hydrogel obtain a high conductivity of 5.2 S/m. Guo et al. [58] introduced LiCl and KOH into the poly(amidoxime)/polyethyleneimine (PAO/PEL) hydrogel and achieved ionic conductivity of 19.1 S/m and 22.35 S/m, respectively. The new ion channels introduced by LiCl and the synergistic effect of metal ions with PAO/PEI significantly improved the ionic conductivity of the hydrogel.

Inorganic salts can provide excellent conductivity to hydrogels, but the addition of large concentrations of inorganic salts can be disruptive to the physical cross-linking of the hydrogel [59,60]. Compared to adding inorganic salts to hydrogels, ionic liquids do not damage the internal structure of hydrogels and provide better biocompatibility. Therefore, ionic conductivity of hydrogels can also be achieved by adding ionic liquids, i.e., ionogel [61,62]. Ionogels have good electrical conductivity and are currently used in ion-gated transistors, strain sensors, etc. [63,64]. Although ionic liquids can provide excellent electrical conductivity to hydrogels, the mechanical properties of the hydrogels prepared by them are poor. To solve this issue, He et al. [65] increased the maximum tensile strength of the ionogels to 0.14 MPa by adding physical cross-linking to the hydrogel using the addition of XLG. They used [EMIM][Cl] ionic liquids to enhance the electrical conductivity of hydrogels and prepared poly(acrylamide-co-2-hydroxyethyl acrylate)/XLG ionogels by UV photopolymerization (Figure 3c).

### 2.2. Mechanical Properties 

To improve the durability of hydrogel-based bioelectronics, the mechanical properties of hydrogels are critical. The mechanical properties of hydrogels are often evaluated by the strength and toughness of the material [66]. Meanwhile, as a common material for wearable electronic and motion monitoring devices [67], it will undergo varying degrees of deformation due to human movements when the device is attached to the human body. Therefore, the equipment needs to have a certain degree of ductility. In this context, the fabrication of conductive hydrogels with excellent mechanical properties are of great importance. Hydrogels are mostly made from solutions of molecular building blocks. After cross-linking, these blocks are transformed into a permeable network [68]. The hydrogels formed under this method often suffer from problems such as inhomogeneous microstructures, which affect their mechanical properties.

**Figure 4:**
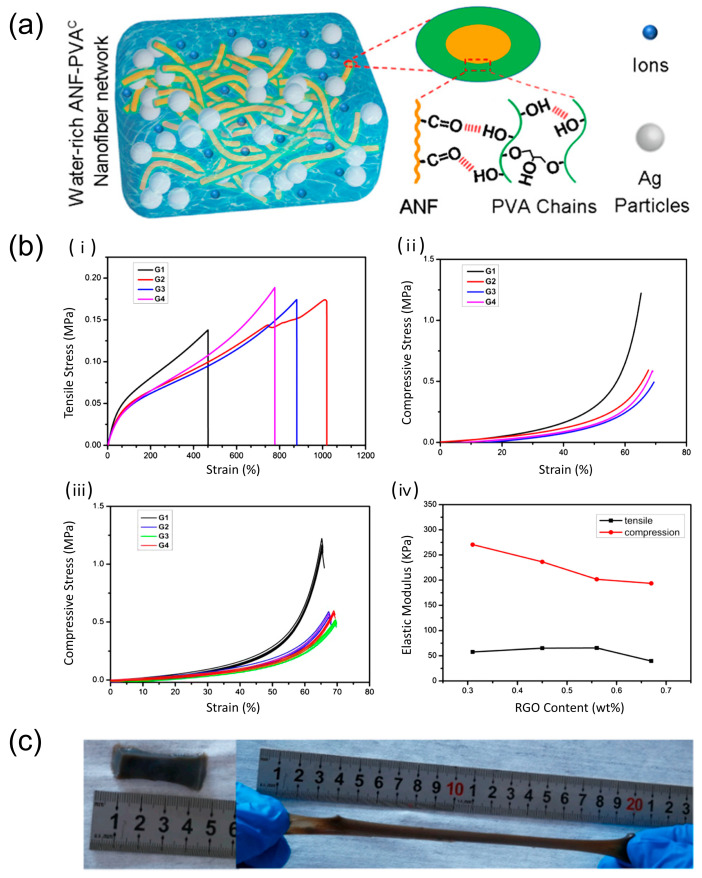
Mechanism and tests of mechanical properties. (**a**) Schematic diagram of a network with both covalent and noncovalent cross-linking. With such cross-linked networks, the mechanical properties can be enhanced. Reprint with permission from Ref. [69]. Copyright 2021 American Chemical Society. (**b**) Mechanical properties of the hydrogel: (**i**) tensile stress–strain, (**ii**) compressive stress–strain for one time and (**iii**) for five times, (**iv**) elastic modulus. Reprint with permission from Ref. [74]. Copyright 2019 John Wiley & Sons. (**c**) Schematic diagram of the tensile property of the hydrogel. With excellent mechanical property, the hydrogel can be stretched to over four times of its original length without any break. Reprint with permission from Ref. [75]. Copyright 2021 Elsevier.

Integrating special materials into the hydrogel structure can combine the advantages into the hydrogel, thus it can improve the mechanical properties. Special components such as aramid nanofibers are added to form cross-linking networks to enhance the mechanical properties (Figure 4a) [69]. In addition, spiropyran (SP), a mechanophore that can respond to multiple stimuli [70,71,72], has been found to effectively enhance the mechanical properties of hydrogel when it participates in the preparation of the hydrogel [73]. The research incorporates SP into polymer networks to develop SP-cross-linked poly(AM-co-MA/SP) (polyacrylamide-co-methyl acrylate/spiropyran) hydrogels. In the process of gelation, SP can form stable cross-links with hydrophobic methyl acrylate. Meanwhile, hydrophilic PAAm chains were also attached to SP-cross-linked PMA microspheres to form the network of the poly(AM-co-MA/SP) hydrogel. Thus, under specific conditions, the tensile stress can reach 1.45 MPa, and fracture energy can reach 7300 J m^−2^. Incorporating nanoparticles is another option. As Figure 4b shows, graphene nanocomposite hydrogels exhibit nice performance in various mechanical properties [74].

In addition to using multi-component particles, the mechanical properties of hydrogels can also be enhanced by the addition of substances such as silk fibroin (SF). Inert silk fibrillar protein nanofibers (SNFs) were introduced into the enzymatic cross-linking system of regenerated silk fibrillar protein (RSF), and the mechanical properties were improved by embedding the inert SNFs into the RSF hydrogel matrix after the RSF cross-linking reaction. Adding to the polar groups modified cellulose nanocrystal (CNC) is another solution [75]. The hydrogel can be stretched to its fourfold length without fracture (Figure 4c). Another option for improving the mechanical properties of hydrogels is the addition of monodisperse colloidal particles. H. Dehne et al. [76] investigated a hybrid gel that combines a PAAm hydrogel with a DNA-coated colloid (DNAcc) to improve the mechanical properties of the hydrogel. It has been suggested that polymer chains, after adhering to the colloid surface, form polymer coronas around the colloid, thereby strengthening the interactions between the colloids and improving their strength [77].

There are many ways to achieve dynamic mechanical properties, where the shape of the hydrogel changes somewhat in response to some stimulus [78]. The ductility of hydrogels can be achieved by gradient structures. Whether the polymer chains have a gradient distribution or the filler exhibits a gradient distribution, hydrogels are able to effectively produce complex deformations [79]. Akashi et al. used electrophoresis and photopolymerization to prepare thermoresponsive poly(N-isopropylacrylamide) (PNIPAAm) hydrogels, which were able to deform with temperature changes [80]. Liu et al. introduced magnetic nanoparticles into the hydrogels and prepared composite hydrogels that were able to deform with magnetic field changes [78].

### 2.3. Self-Healing

**Figure 5:**
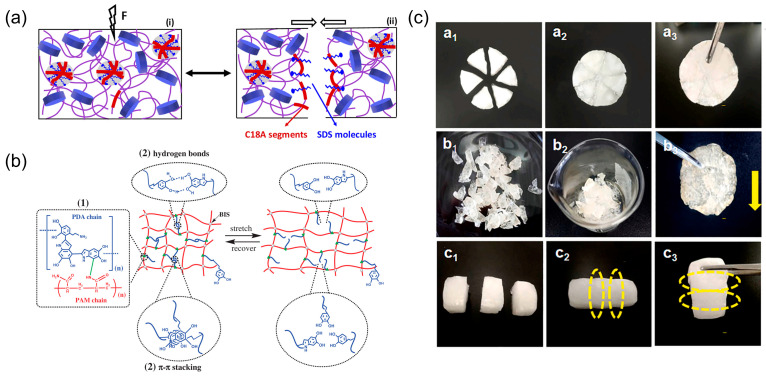
Mechanism and tests of self-healing. (**a**) Schematic diagram of a kind of self-healing mechanisms of hydrogels. Reprint with permission from Ref. [102]. Copyright 2021 John Wiley & Sons. (**b**) Schematic diagram of the structure of the polydopamine–polyacrylamide (PDA–PAAm) hydrogel. Catechol groups of the PDA chains form two kinds of reversible noncovalent bonds: π–π stacking and hydrogen bonds. Reprint with permission from Ref. [89]. Copyright 2007 The American Association for the Advancement of Science. (**c**) Self-healing tests of PAMSA-15 hydrogel samples. This shows that this kind of hydrogel with different shapes can heal at room temperature. Reprint with permission from Ref. [91]. Copyright 2020 Elsevier.

The self-healing property of hydrogel is one of the remarkable factors for achieving the stable functionality of the device’s period of service. It refers to the ability of a material to heal damages and restore itself to normality intrinsically and automatically [81]. One of the self-healing mechanisms is shown in Figure 5a. This figure shows that when the hydrogel has hydrogen bonds, the process of self-healing is achieved by the generation of a 3D physical network through hydrological interactions. It can be used as an artificial implant in clinical applications such as an artificial meniscus [82]. For the property of self-healing, when it meets some crack or flow, it can recover itself for longer service life, which relieves the pain of the patient by avoiding repetitive surgery.

From the perspective of the self-healing hydrogels to be cross-linked, there are two bonds: covalent bonds and non-covalent bonds. Most hydrogels do not readily reform covalent sacrificial bonds when they undergo large deformations or rupture after cyclic loading [83]. The number and the type of the linkages decide the degree of the self-healing. In order to achieve greater self-healing, many methods have been proposed for improving this performance from various aspects such as different bonds, materials, and so on.

Among these methods, there is a very special category that is inspired by organisms in nature, including mussels, barnacles, and sandcastle worms [84,85,86]. Mussels are bivalve mollusks living in saltwater or freshwater. In nature, mussels can form hard adherent plaques by secreting adhesion proteins that adhere tightly to foreign surfaces in seawater [84]. Taking inspiration from the mussel, many hydrogels with high self-healing performance have been developed successfully. Similar to its protein, as shown in Figure 5b, when the catechol-based dynamic bond constructed in the hydrogel can be broken easily and can also be reconstructed to the incipiency, the hydrogel can obtain the property of self-healing. Similar to the adhesive protein of mussels, polydopamine (PDA) can adhere to many kinds of surfaces. In the acidic and reducing environment, dopa can maintain stability by the isolated dopa(3,4-dihydroxy-phenyl-L-alanine)-containing adhesive proteins [87]. Back in 2007, Lee et al. found a method that can prepare multifunctional polymer coatings in simple dip coatings in aqueous dopamine solutions [88]. The proposal lays the foundation for the subsequent development of hydrogels inspired by mussels.

In 2017, Han et al. proposed a new hydrogel consisting of a single network formed by polydopamine–polyacrylamide (PDA–PAAm) [89]. Since PDA has highly catecholic groups, this hydrogel can self-heal in some time. When the hydrogel was divided into two parts and then the pieces contacted each other, after 2 h, they could rejoin at room temperature. In addition to dopamine, these kinds of catechol-based derivatives are also inspired by the mussel: 3,4-dihydroxyphenylalanine (DOPA), norepinephrine, gallic acid, and tannic acid (TA). Based on PVA, hydrogels can be prepared to have low permeability and self-healing properties at the same time. This material can be used to make artificial cartilage, which is one of the best materials for artificial joint repair [90]. When sodium alginate (SA) and PAAm are combined, the hydrogel has 99% self-healing properties by promoting the self-assembly property of hydrogen bonds (HBs) in the PAAm matrix [91]. Its test diagrams are shown in Figure 5c.

With the exception of structures such as organisms in nature, self-healing can be realized by chemical methods. When the cross-linking mechanism is different, the stimulus that is required to achieve self-healing is different. According to the study, it is known that the self-healing property can be activated by heat [92], light [93], enzymes [94], and other external stimuli. Marie et al. came up with a synthetic self-healing hydrogel system, which can respond to multiple pH values [95]. In this system, cross-linking is related to the pH. When the pH is lower than the pI value of the polymer, the polymer has a high charge, which promotes the formation of hydrogels. Therefore, as pH increases from an acidic to a basic value, the hydrogels can turn to the quickly self-healing ones, which also have high strength. In addition to pH, self-healing hydrogels can be activated by light. These hydrogels are transparent in visible light. Meanwhile, they can absorb the light of other frequencies, such as ultraviolet (UV), infrared (IR), and microwave [96]. Two parts of these hydrogels can rejoin in just 12 s at the indoor temperature.

In addition to synthetic hydrogels, supramolecular hydrogels have been a hot research topic in recent years. According to their formation mechanism, supramolecular materials go through one-dimensional assembly of molecular stacking motifs, which can also be prepared by polymer precursors cross-linking with the molecular recognition motifs [97]. They are three-dimensional (3D) hydrophilic cross-linked polymers with high performance regarding the self-healing property. Supramolecular hydrogels are formed through supramolecular chemical pathways (such as self-assembly, host–guest combination, molecular recognition). They have a 3D cross-linked scaffold with high loading capacity, significant biocompatibility, and biodegradability [98]. Because of the dynamic/switchable nature of the supramolecular cross-linked network, self-healing is possible [99]. For this reason, supramolecular hydrogels are widely used in medical diagnosis and treatment [100,101].

### 2.4. Adhesion

Hydrogel-based bioelectronics often need to be adhered to the surface of human skin to achieve health monitoring directly. Since the main component in hydrogels is water, polymers make up only a small part. In addition, water can easily lead to a small transmitting force with the change in the structure of adjacent molecules [23]. This causes a challenge to the adhesion of hydrogels. However, maintaining the relative stability between the sensor and the human body during measurement can improve the accuracy of the measurement results. Therefore, if the sensing interface itself is adhesive, this will bring great convenience and benefit to the development of the device. The hydrogel-based health monitoring device is equipped with these functions. Furthermore, stronger adhesion can be achieved by combining the supramolecular synergy of chemical bonds, topology, and mechanics.

**Figure 6:**
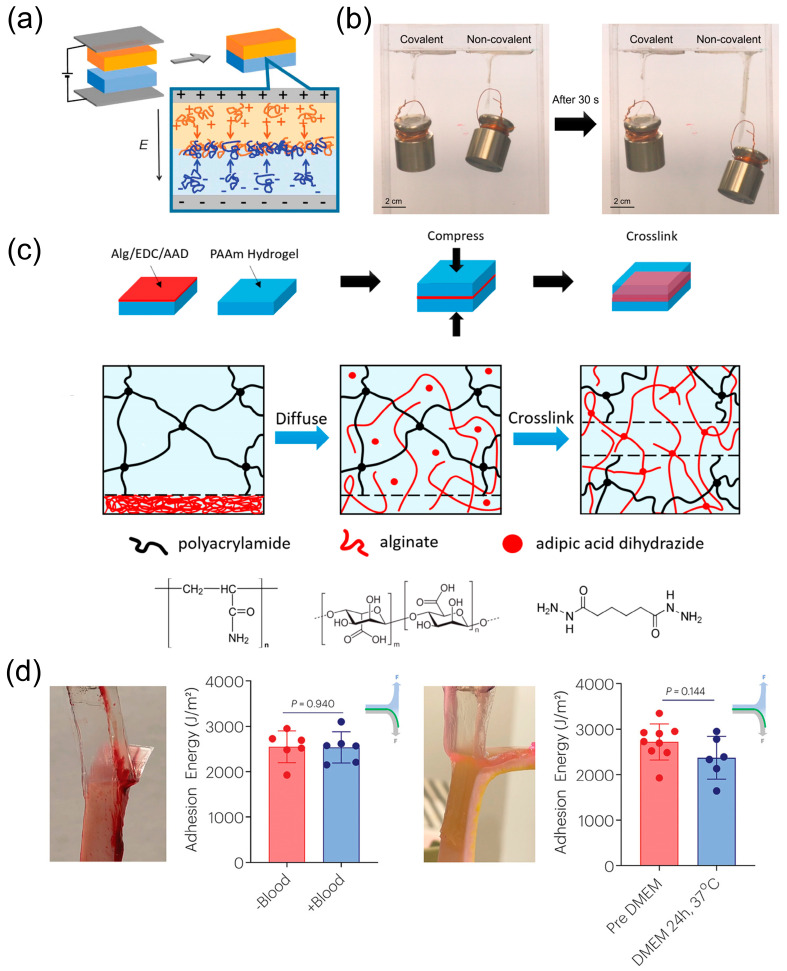
Mechanism and tests of adhesion. (**a**) Schematic diagram of the electrophoresis of cationic and anionic hydrogels. Cationic polymers are shown in orange, while anionic polymers are shown in blue. Reprint with permission from Ref. [103]. Copyright 2016 The Society of Polymer Science, Japan. (**b**) Comparison of the stability of covalent and non-covalent topological adhesion. The environment is a phosphate buffered saline (PBS) solution. After 30 s, covalent topological adhesion remained stationary, while non-covalent topological adhesion detached at a rate of ~1 mm s^−1^. Reprint with permission from Ref. [107]. Copyright 2019 American Chemical Society. (**c**) Covalent topological adhesion of two PAAm hydrogels. Reprint with permission from Ref. [107]. Copyright 2019 American Chemical Society. (**d**) For blood and incubation in Dulbecco’s Modified Eagle Medium (DMEM), chitosan non-covalent adhesion is steady. Reprint with permission from Ref. [108]. Copyright 2022 John Wiley & Sons.

Chemical bonds are formed commonly to achieve adhesion. The electrophoretic technique is a commonly used chemical method [103]. The schematic diagram is shown in Figure 6a. During the electrophoresis process, the cationic polymer moves to the cathode and the anionic polymer moves to the anode. Then, the polyions diffuse within the gel and form a polyionic complex at the interface of the hydrogel to achieve adhesion. Li et al. [104] developed a chitosan hydrogel membrane (CHM), a highly adhesive membrane based on chitosan. When negatively charged in alginate solution, chitosan can have interactions with it and produce a water-insoluble membrane. This gives the possibility of the presence of adhesion in the CHM. In addition, Liu et al. have successfully explored bioinspired adhesion hydrogels from separate nucleobases (adenine, thymine, guanine, cytosine, and uracil) treated in DNA and RNA [105]. Each base is independently introduced into the PAAm, which allows the hydrogel to possess hydrophobic interactions, metal complexation, π-π stacking, and other possible reactions, which lead to adhesion behavior. The DNA/RNA-based hydrogel not only has strong adhesion, but also will not cause harm to the human body because it is an inherent molecule in the human body.

In addition, a topological linkage is formed by a network of two polymer chains without functional groups. At the molecular scale, it is much like being stitched together [106]. The topology is capable of forming stronger cross-links. When water drops on it, it does not have an effect on its adhesion. The structure can be permanent, temporary, or formed under special conditions. Hydrogen bonds, covalent bonds, etc., can be used to develop topological linkage stitched networks. Covalent topological connections are sometimes more stable than non-covalent connections [107]. As shown in Figure 6b, in 30 s, the covalent topological adhesions do not divide, while the hydrogel with non-covalent topological adhesions divide at about 1 mm s^−1^. Figure 6c shows one of the applications of covalent topology. Two PAAm hydrogels are linked by covalent topology. To achieve this, researchers mixed alginate, adipic acid dihydrazide (AAD), N-hydroxysuccinimide (NHS), and 1-ethyl-3-(3(dimethylamino)propyl)carbodiimide (EDC) in an aqueous solution, and then the above aqueous solution was uniformly covered onto a 500 μm-layer of PAAm hydrogel. Then, this hydrogel was given a compressive strain of about 5.5%. Because of EDC and NHS, the alginate chains are covalently cross-linked by AAD and form a covalent network with the PAAm network topology entangled in situ.

However, regardless of the covalent or non-covalent bonds, there may be problems such as unstable, weak, and slow adhesion. To address the above problems, Hamza et al. [108] found that if the hydrogel dissipative matrix can combine with the pH-responsive bridging chitosan polymer chain, strong adhesion can be achieved in a short time. It can be seen through Figure 6d that chitosan non-covalent adhesion in Dulbecco’s Modified Eagle Medium (DMEM) is steady.

Hydrogels with only a single connection tend to have poor adhesion. In order to achieve strong adhesion, multiple attachment methods are used. Polyvinyl pyrrolidone-PAA (PVP-PAA) and PVA-PAAm hydrogels can achieve the function mentioned above [109]. The strong adhesion of these hydrogels is achieved through the following mechanisms: bonding achieved by the two kinds of polymer, one is pre-existing polymer (PVP or PVA), one is in situ polymerized polymer (PAA or PAAm). The electrostatic interactions generated by the contact of two interfaces under pressure and the pre-complexation of PAA with Fe^3+^ should take place at conditions with specific pH. In addition to the above bonds, polymer complexes, ionic bonds, dipole–dipole interactions, and other bonds can enhance the adhesion properties.

### 2.5. Biocompatibility

**Figure 7:**
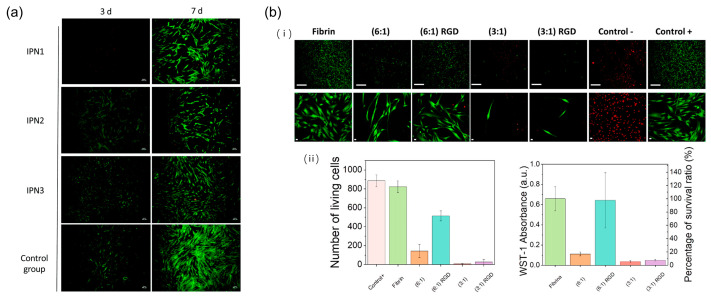
Display of biocompatibility. (**a**) Comparison of the situation of human mesenchymal stem cells (hMSCs) after 3 and 7 days of exposure to interpenetrating network (IPN) hydrogels. Green fluorescence represents living cells, while red represents dead cells. Scale bar = 100 μm. Reprint with permission from Ref. [116]. Copyright 2020 The Royal Society of Chemistry. (**b**) Tests of the hydrogel biocompatibility. (i) Microscopic images of the living state of cells after contact with the hydrogel for 48 h. (ii) With three peptides (Fmo-FF, Fmoc-RGD, and fibrinogen) co-assembled together, the hydrogel’s impact on cell viability can be significantly reduced. Reprint with permission from Ref. [122]. Copyright 2023 American Chemical Society.

The biocompatibility of hydrogel devices has become a key issue because problems such as immune reactions, breathability, and skin-attachable properties can affect the accuracy of monitoring [110,111,112]. At the same time, if the monitor appears in the form of a probe, the interface between the material and tissue will form a complex surface after the device is implanted [113]. The probe surface will be coated with substances such as fibrinogen, IgG, and fibronectin. It is generally believed that protein adhesion is the first step in causing infection in organisms [114]. After adhesion, the protein denatures. The organism immediately begins a series of immune reactions such as neutrophil infiltration (acute inflammation) and recruitment of macrophages and monocytes (chronic activation) [115]. Over time, there will be more and more immune reaction products such as proteins attached to the device. As shown in Figure 7a, after a period of implantation of the hydrogel, the biological tissue develops an immune response. The picture uses live/dead staining as a method to visually reflect the biocompatibility of the hydrogel. If biocompatibility is good, there are more live cells [116]. Not only does it affect the normal operation of health monitoring equipment, but it also leads to an increase in the accumulation of metabolites in the human body, making it difficult to carry out normal transportation with the outside body [117]. Therefore, improving the biocompatibility of the device is crucial.

In the applications, many biomaterials demonstrate exceptional biocompatibility. Peptide-based hydrogel is one of the biodegradable biocompatible biomaterials. The peptides are naturally biocompatible and have special advantages such as multiple functional groups and antibacterial activity, so problems such as inflammation and hyperpigmentation are virtually non-existent when the hydrogel is implanted in the body. After comparative experiments with normal human cells, the results show that under certain conditions, peptide-based hydrogels can achieve the same function without causing damage to the cells [110]. Composite fibrin with some specific molecules to manufacture the short-peptide supramolecular hydrogels can optimize the biocompatibility. As Figure 7b shows, with appropriate proportions of the three peptides (Fmoc-FF, Fmoc-RGD, and fibrinogen) co-assembled together, the impact on cell viability can be significantly reduced, which means the biocompatibility of the hydrogel has been improved. Pectin is another natural polymer with a high degree of biocompatibility. Markov et al. have prepared a pectin hydrogel [117]. In testing, experiments were focused on its hemolytic levels. In clinical practice, in the event of hemolysis, serious consequences such as anemia, impaired kidney function, and jaundice may eventually occur. According to the test results, the above pectin hydrogels do not affect the red blood cell membrane and have good hemo-compatibility.

Except for natural biomaterials, many synthetic materials also have excellent biocompatibility properties. They are widely used in artificial stents and intraocular cataract lenses (IOLs). Pars Plana Vitrectomy (PPV) has evolved into the most effective tool in the treatment of conditions such as cataracts and retinal detachment [118]. Although artificial vitreous substitutes based on collagen and hyaluronic acid also fulfil the established functions, problems such as low degradation rates and low viscosity pose certain pitfalls. Based on this, a hydrogel-based alternative to vitreous was created. The vitreous substitutes mentioned above remain stable in the body for more than two months. Lee et al. [119] developed a poly hydrogel, which has an outer wall with a high-modulus polyethylene glycol diacrylate (PEGDA) structure, that adheres to the interface in a shape similar to an octopus suction cup. The internal material is low-modulus poly(N-isopropylacrylamide) (pNIPAM), whose volume can vary with temperature. By placing cells in an environment that mimics pNIPAM and PEGDA hydrogels, the cells grew well. Therefore, it can be seen that this kind of hydrogel has high biocompatibility.

In addition to the biocompatibility of the hydrogel by itself, its biocompatibility is also crucial when it is used in health detection devices. Hua et al. [120] studied a xanthan gum-based hydrogel which can show excellent biocompatibility in a soft conductive device. In the research, they use fibroblast cells (L929) to study the cytotoxicity of the hydrogel. After the test, the sample did not lose more viability with the existence of the hydrogel, which represents its great biocompatibility. Recently, a PAAm nanocomposite hydrogel has demonstrated its excellent biocompatibility in flexible electronic devices [121]. The hydrogel adopts polydopamine-modified carbon nanotubes (PDA@CNTs) into the preparation in order to achieve superior biocompatibility for long term health monitoring. This research has shown great application prospects in human–computer interaction, medical communication, and other fields with the help of biocompatible hydrogels.

## 3. Hydrogel-Based Bioelectronics for Health Monitoring

Compared with traditional health monitoring devices or wearable sensors, hydrogel-based bioelectronics give the devices better flexibility and provide a more comfortable human–machine interface [17]. The mechanical properties, self-healing, and biocompatibility of hydrogels help to further improve the wearing comfort and signal-to-noise ratio of health monitoring devices [123]. Based on their location and depth of penetration into the skin, hydrogel-based bioelectronics can be classified as skin-attachable, implantable, or semi-implantable bioelectronics.

### 3.1. Skin-Attachable Hydrogel-Based Bioelectronics

Attached to the skin surface is a common form of bioelectronic used for health monitoring. Compared to implantable and semi-implantable bioelectronics, skin-attachable bioelectronics do not need to penetrate human skin and do not cause harm to the body. Due to its tunable conductivity, the hydrogel can convert mechanical, thermal, and other signals into electrical signals or directly monitor bioelectrical signals, enabling high-quality health monitoring on the skin surface.

#### 3.1.1. Bioelectronics for Physical Indicators

Physical indicators refer to human motion signals, humidity, bioelectrical signals, etc. Hydrogels have a small Young’s modulus and can achieve large stretching without fracture, which makes them suitable for preparing stress–strain sensors for human motion, pulse, etc., to achieve health monitoring [124,125]. Hydrogel stress–strain sensors can be based on a variety of monitoring principles such as resistive, capacitive, piezoelectric, and triboelectric, among which resistive and capacitive sensors have been researched extensively [126,127,128,129]. Under stress or strain, the hydrogel network is squeezed and the hydrogel conductivity or the capacitance of the sensor changes accordingly to achieve stress–strain monitoring.

**Figure 8:**
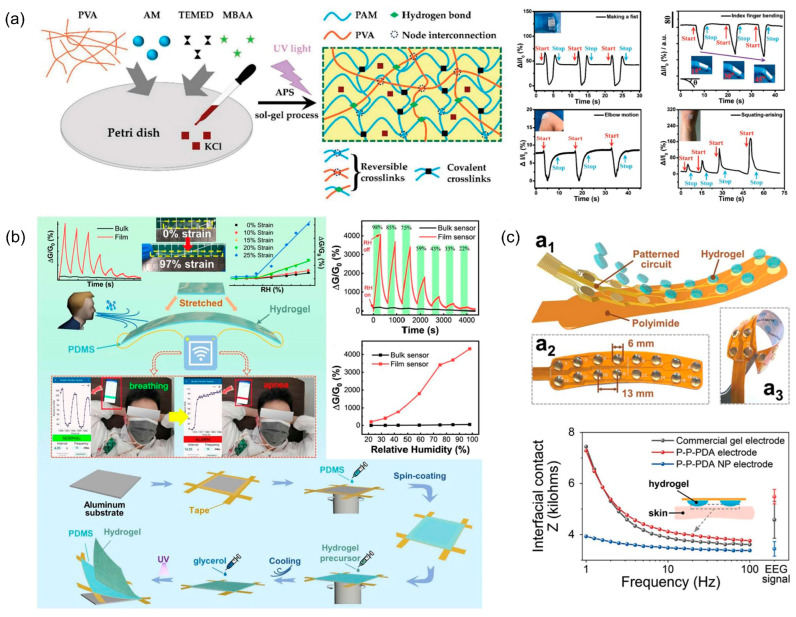
Skin-attachable hydrogel-based bioelectronics for physical indicators. (**a**) Schematic diagram of the binary networked hydrogel and the response of large-scale human motions monitoring. Reprint with permission from Ref. [130]. Copyright 2018 John Wiley & Sons. (**b**) Schematic diagram of the stretchable hydrogel film humidity sensor. Reprint with permission from Ref. [132]. Copyright 2022 Springer Nature. (**c**) Structure and performance of the PVA and polyvinyl pyrrolidone (PVP) hydrogel electrode. Reprint with permission from Ref. [138]. Copyright 2023 John Wiley & Sons.

The monitoring of human motion by hydrogels is based on the conductivity, so the sensitivity of hydrogel stress–strain sensors is closely related to the conductive filler and microstructure of hydrogels. Methods such as forming microstructures on the hydrogel surface or in the dielectric layer can usually be used to improve the sensitivity of the sensor. Ge et al. [130] prepared high tensile (>500%) and high sensitivity (0.05 kPa^−1^ for 0–3.27 kPa) pressure sensors by forming microstructures on the surface of PAAm and PVA double-network hydrogels (Figure 8a). The folded microstructure on the hydrogel surface increases the contact area of the sensor, enabling the monitoring of small physiological activities. The homogeneous self-patterned grooves formed on the surface during the preparation of the hydrogel by solvent evaporation and the reversible physical cross-linking bonds formed by strong hydrogen bonding between PAAm and PVA substantially improved the tensile properties and sensitivity of the hydrogel. Wei et al. [131] fabricated a hydrogel strain sensor that can operate in resistance–capacitance dual mode based on a hydrogel prepared by combining CNTs, polyacrylic acid, and sodium alginate with a chelate of calcium ions. The sensor uses a polyethylene layer as the dielectric layer, and the prepared hydrogel is printed in a grid to connect the electrodes and then encapsulated with polyethylene to prevent dehydration of the hydrogel, which can respond to both resistive and capacitive changes.

The conductivity of hydrogels does not only change due to deformation. Under different humidity conditions, the hydrophilic groups in hydrogels combine with water molecules to different degrees, causing the conductivity of hydrogels to change accordingly. Therefore, the conductivity of hydrogels is directly related to their water content. Based on the sensitivity of hydrogel conductivity to humidity, the humidity sensor can be prepared using hydrogels for detecting human respiration, body surface humidity, etc. Liang et al. [132] proposed a high-sensitivity hydrogel humidity sensor by introducing a cassava powder cross-linked network in PAAm, which can be effectively monitored by sensing and affixing to the inside of a mask (Figure 8b). In order to achieve stable and accurate monitoring of humidity, hydrogel-based bioelectronics especially need to consider their water retention to avoid dehydration under low-humidity conditions. Bai et al. [133] synthesized an organic hydrogel-based bioelectronic based on natural skin using natural skin extracted from goats as the substrate of the hydrogel and introduced a mixture formed by betaine, silver nanoparticles, sodium chloride, and glycerol/water binary solvent. Inorganic salts and metal particles provided excellent electrical conductivity to the hydrogel, and the introduced glycerol/water binary solvent and betaine slowed down the drying rate of the hydrogel. The mechanical stability provided by the natural skin skeleton and the antibacterial and moisturizing properties brought by the filler enable the bioelectronic to be attached to human skin to effectively sense environmental humidity changes.

Hydrogels can be used to prepare flexible electrodes for bioelectrical signal acquisition, such as electroencephalogram (EEG) and electrocardiogram (ECG), due to their softness and conductivity [134,135]. Clinically, rigid electrodes are usually used and coated with conductive gel to ensure contact between electrodes and tissues and to reduce contact impedance [136]. Conductive gels require frequent replacement and can make the acquisition of electrical signals cumbersome due to their poor biocompatibility and stability. The low Young’s modulus and high biocompatibility of the hydrogel can solve problems such as allergy brought by traditional electrodes to a certain extent, but it is still necessary to further improve the conductivity stability and lifetime of hydrogels and reduce the contact resistance with tissues. Inspired by the skin sebum membrane, Leng et al. [137] incorporated bovine serum albumin with proton-conducting properties into the hydrogel and used glycerol, which is naturally present on the skin surface, as an artificial sebum membrane to prevent dehydration of the hydrogel to maintain its electrical conductivity. Han et al. [138] improved the electrical conductivity of hydrogels by incorporating polydopamine particles obtained by free radical oxidative degradation treatment into PVA and polyvinylpyrrolidone hydrogels with multifunctional nanoparticle enhancement effects and made the hydrogels highly flexible and adherent (Figure 8c). The hydrogel electrode has low contact impedance (3–4 kΩ), and high-quality EEG signals can be collected stably under sweating conditions.

In addition to directly using the hydrogel’s response to external stimuli and changes in electrical conductivity to achieve health monitoring, the biocompatibility of the hydrogel can also be used as a friendly skin contact interface to provide greater comfort for skin-attachable health monitoring [112,139]. Zhao et al. [26] reported a bioadhesive hydrogel ultrasound patch that can perform 48 h internal continuous imaging of organs such as blood vessels and heart. By combining the advantages of elastomers and hydrogels, the chitosan-PAAm interpenetrating network hydrogel is encapsulated in a thin elastomeric membrane that prevents hydrogel dehydration and provides comfortable skin contact on the coupling agent surface. The hydrogel imaging platform can detect changes in heart size during human exercise and in the size of the stomach of volunteers after drinking juice.

#### 3.1.2. Bioelectronics for Chemical Indicators

Chemical indicators in the human body are usually obtained by detecting the content of chemical molecules in body fluids, commonly known as sweat and tears [140]. Hydrogel-based sweat bioelectronics have been heavily investigated due to the ease of access to sweat [141]. Human sweat contains biomarkers such as glucose, electrolytes, lactate, and hormones [142]. Changes in the concentrations of these biomarkers in sweat are closely related to human activity, and monitoring the levels of biomarkers in sweat can serve the purpose of health monitoring and aid in diagnosis [143,144,145]. Qin et al. [146] reported a self-powered sweat sensor based on cellulose nanocomposite hydrogel electrodes. A conductive hydrogel electrode with rapid self-healing and high stretchability was prepared by in situ polymerization of 2,2,6,6-tetramethylpiperidine-1-oxyl radical (TEMPO)-oxidized cellulose nanofibrils (TOCNF)/PANI nanocomposite made from PANI on TOCNF and cross-linked with PVA/borax. The electrode can achieve 95% self-healing efficiency within 10 s. Its high conductivity (0.6 S/m) allows further preparation of the triboelectric nanogenerator for self-powered sweat sensing, avoiding the use of rigid power supply devices.

**Figure 9:**
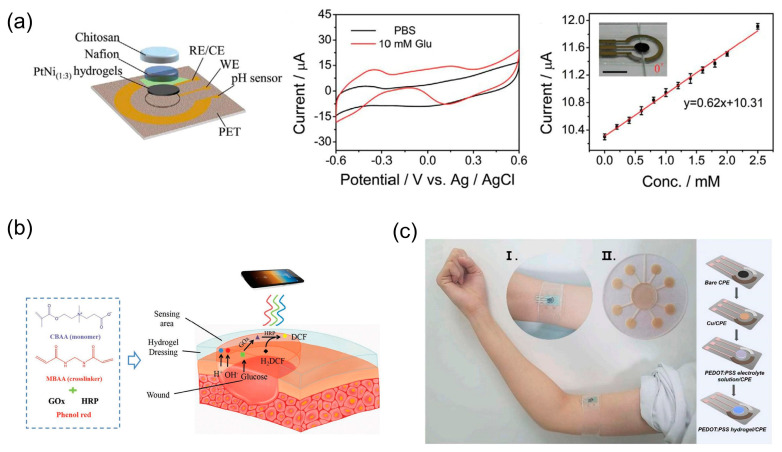
Skin-attachable hydrogel-based bioelectronics for chemical indicators. (**a**) Schematic diagram and performance of the PtNi_(1:3)_ hydrogel-based sensing chip. Reprint with permission from Ref. [149]. Copyright 2023 John Wiley & Sons. (**b**) Schematic diagram of the poly-carboxybetaine hydrogel dressing that can detect pH and glucose at the wound site. Reprint with permission from Ref. [151]. Copyright 2019 John Wiley & Sons. (**c**) Schematic diagram of the preparation process of the poly(3,4-ethylenedioxythiophene):poly(styrene sulfonate) (PEDOT:PSS) hydrogel-based sensing platform and skin-attachable bioelectronic configuration on the upper arm. Reprint with permission from Ref. [154]. Copyright 2021 Elsevier.

In recent years, the incidence of diabetes has gradually increased due to changes in people’s lifestyles. Diabetes brings several complications, and high blood glucose may also lead to an increased risk of cancer in diabetic patients [147,148]. In addition, other complications caused by hyperglycemia need to be taken into account. In diabetic patients, hyperglycemia can lead to poor circulation and impede wound healing, so glucose is also an important indicator to monitor in wound healing. Thus, convenient and rapid glucose monitoring methods need to be researched to address the need for glucose monitoring. The recognition of biomolecules in general requires the immobilization of recognition sites such as enzymes on sensors, but the stability of bioactive enzymes hinders the stable and accurate monitoring of biomolecules, so it is important to study the immobilization methods of enzymes on sensors or non-enzymatic sensors. Li et al. [149] synthesized a three-dimensional dual-structured PtNi hydrogel interconnected with a network of PtNi nanowires and Ni(OH)_2_ nanosheets for non-enzymatic glucose sensing (Figure 9a). The synergistic effect of Pt and Ni in the PtNi dual-structured hydrogel synthesized by co-reduction of a mixture of chloroplatinic acid and nickel chloride using sodium borohydride at room temperature significantly enhances its electrocatalytic activity and promotes glucose oxidation. Hydrogel-based bioelectronics also enable simultaneous monitoring of multiple parameters for wound monitoring and avoid the risk of infection associated with frequent dressing changes [150]. Zhu et al. [151] reported a hydrogel dressing that can detect pH and glucose at the wound site using optical methods (Figure 9b). The stability of glucose oxidase and horseradish peroxidase and the sensitivity of the sensor were improved by exploiting the superhydrophilicity of amphiphilic ionic groups and encapsulating the enzyme molecules into a poly-carboxybetaine hydrogel, avoiding the interference of other biomolecules on the enzyme binding site. The fluorescent products generated by oxidation can be collected and extracted using a smartphone, enabling accurate analysis of wound pH and glucose concentration.

In addition to monitoring glucose, hydrogel-based bioelectronics can also monitor other biomolecules such as uric acid and cholesterol [152,153]. Traditionally, uric acid is monitored at home by testing blood from the fingertip, which requires frequent and cumbersome finger pricks. Xu et al. [154] reported an electrochemical sensor based on a poly(3,4-ethylenedioxythiophene):poly(styrene sulfonate) (PEDOT:PSS) hydrogel (Figure 9c). The good electrical conductivity and large electroactive surface area of PEDOT:PSS hydrogel make it a good electrochemical catalyst for the oxidation of uric acid. By designing a microfluidic device to collect sweat, this hydrogel-based bioelectronic can monitor the uric acid content in it.

#### 3.1.3. Multifunctional Skin-Attachable Bioelectronics

The implementation of multifunctional and multi-indicator health monitoring or their combination with therapeutic functions on a single hydrogel bioelectronic is more suitable to meet the needs of personalized medicine. The tunable nature and multi-parameter sensitivity of hydrogels also give hydrogel-based skin-on bioelectronics more application possibilities. Li et al. [155] prepared hydrogel–paper electrodes by self-assembling highly conductive PEDOT:PSS hydrogels on paper fibers. By further preparing a low-resistance ECG electrode and a glucose sensor and integrating them with the FPCB, simultaneous acquisition of bioelectrical and biochemical signals can be achieved. Liu et al. [156] reported a multifunctional bioelectronic based on polyurethane elastomer that can be used as a stress–strain sensor for human motion monitoring and an electrode to detect ECG and electrooculogram (EOG). Wan et al. [157] introduced antimicrobial Ag nanoparticle-modified MXene nanosheets into a polymer network of guar and phenylboronic acid grafted sodium alginate and prepared MXene-based hydrogels for human electrical signal monitoring. Meanwhile, the antimicrobial and injectable properties make it possible to inject into the wound site to promote wound healing.

### 3.2. Implantable Hydrogel-Based Bioelectronics

Compared to skin-attachable bioelectronics, implantable bioelectronics allow further health monitoring of human internal organs. Implantable medical devices such as cardiac pacemakers and nerve electrodes have been widely used in clinical health monitoring and disease treatment. However, these devices are mostly rigid and cannot fit closely to biological tissues. The inability to produce deformation also makes traditional rigid implantable bioelectronics susceptible to human motion [158]. Since implantable bioelectronics are placed inside the human body, it is also important to consider the histocompatibility of the device to avoid immune rejection [159]. Hydrogels not only have similar properties to biological tissues, such as water content, but their strong adhesion to biological tissues can also further reduce the electrical signal transmission between implantable bioelectronics and tissues, making them ideal interface materials [160,161,162,163].

**Figure 10:**
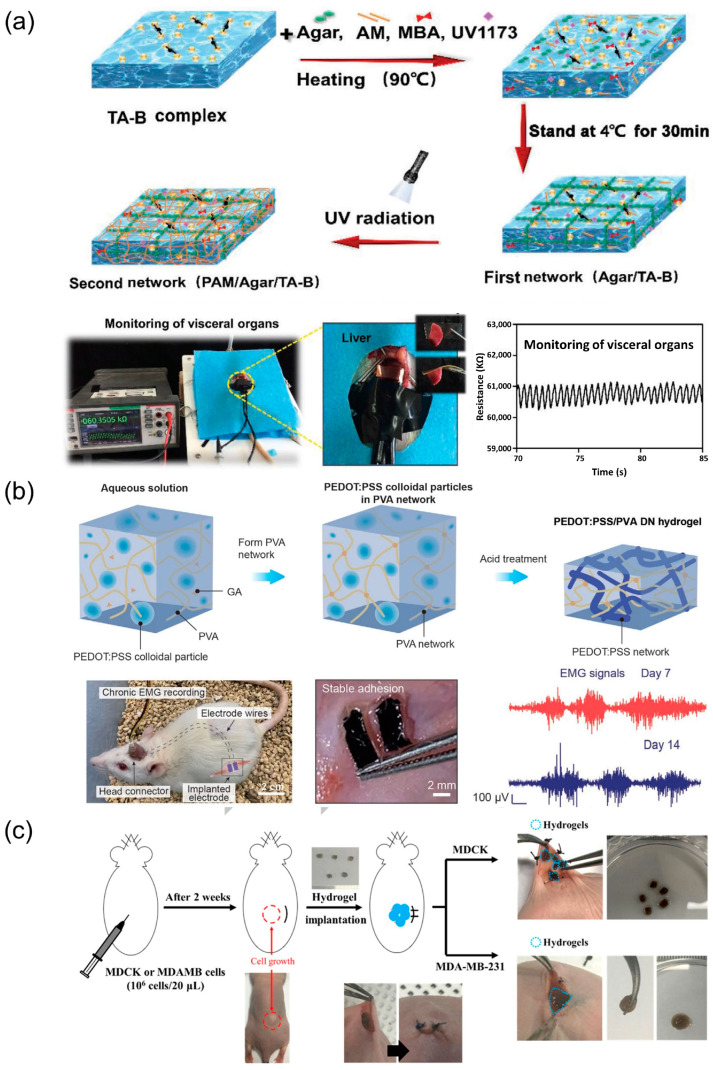
Implantable hydrogel-based bioelectronics for health monitoring. (**a**) Schematic diagram of the synthesis of the PAAm/agarose/tannic acid-borax hydrogel and the record of monitoring a visceral organ. Reprint with permission from Ref. [36]. Copyright 2021 John Wiley & Sons. (**b**) Schematic diagram of the preparation process of the PEDOT:PSS/PVA dual-network hydrogel and the implantation in a rat for chronic electromyogram (EMG) recording. Reprint with permission from Ref. [169]. Copyright 2022 John Wiley & Sons. (**c**) Schematic diagram of in vivo hydrogels in response to cancer cells. Reprint with permission from Ref. [173]. Copyright 2020 American Chemical Society.

Hydrogel-based bioelectronics implanted in tissues need to be considered firstly for material biocompatibility. Since the high water content of hydrogels provides strong conditions for microbial growth, in addition to avoiding interface mismatch, antimicrobial properties need to be considered to avoid bacterial infection during implantation or monitoring to enhance the biocompatibility of hydrogels and prolong the life of the device [164,165,166]. Lei et al. [36] prepared a PAAm/agarose/tannic acid-borax hydrogel for antimicrobial human health monitoring (Figure 10a). Using the sol-gel conversion method, the addition of agarose enhances the mechanical properties of the PAAm hydrogel, and borax and tannic acid have antibacterial properties and further enhance the electrical conductivity of the hydrogel network. The bioelectronics can be used to monitor heartbeat and wound healing and can be implanted in rats to monitor changes in visceral resistance.

Similar to being on the body surface, hydrogels can be fabricated with implantable electrodes to achieve in vivo electrical signal monitoring [167]. This requires a small interface mismatch between the hydrogel electrode and the tissue and a reduction of the interface impedance by appropriate methods. Liu et al. [21] prepared high conductivity (47.4 ± 1.2 S/cm) hydrogel electrodes by removing the ionic liquid from the PEDOT polymer [168] by water exchange. Moreover, the hydrogel with porous structure could be patterned using perfluoropolyether elastomeric photoresist. In addition to reducing the interfacial impedance between hydrogel and tissue, it is still necessary to pay attention to the unification of mechanical and conductive properties of electrodes. Li et al. [169] reported a PEDOT:PSS and PVA dual-network hydrogel electrode meeting the requirements of high conductivity (≈10 S cm^−1^) and large stretching rate (≈150%). The content and homogeneity of PEDOT:PSS were ensured by the densification method with pure acetic acid treatment to cross-link the soft PVA network with the rigid PEDOT:PSS network to form a dual network. The hydrogel was implanted into rats, and the electrodes allowed for long-term stable monitoring of electromyogram (EMG) for 14 days after implantation (Figure 10b). In addition to implantable electrodes, implantable devices also enable the transmission of electrical signals to biological tissue and the active application of electrical stimulation to treat neurological disorders and organ dysfunctions. Elastomers are commonly used in this type of device [170,171]. Hydrogels are also utilized due to their intrinsic advantages [21]. Hu et al. [172] reported an implantable hydrogel cardiac patch for cardiac function repair by using the ionic conductivity of hydrogels to promote the expression of cardiac-related factors and electrical signaling. To ensure the biocompatibility of the device, carboxybetaine methacrylate and hydroxyethyl methacrylate copolymerized into an amphoteric polymer was used to enhance the conductivity of the hydrogel. The patch enables the combination of health monitoring and therapy, showing the prospect of further expansion of hydrogel-based bioelectronics for clinical applications.

In addition to the use of hydrogel electrodes to obtain electrical signals, implantable hydrogel-based bioelectronics can be used to obtain information about biomarkers in body fluids to help the general public understand their health conditions and to help physicians to provide better diagnoses and treatments. Won et al. [173] proposed the incorporation of carbon dots containing diselenide into hydrogels for the detection of glutathione and reactive oxygen species produced by cancer cells (Figure 10c). The hydrogels were prepared by making gelatin react with ureidopyrimidinone via an isocyanate–amine reaction, where both glutathione and reactive oxygen species cleave the diselenide bond and affect the diselenide-mediated self-healing effect of the hydrogels. Kaefer et al. [174] embedded gold nanoparticles in hydrogels to assess tissue concentrations of kanamycin in anesthetized rats using optical methods. The two gold nanorods in the streak containing gold nanoparticles have different plasmon resonance wavelengths, and kanamycin causes a change in the plasmon resonance wavelength. Information on the amount of kanamycin can be obtained by detecting the spectral shift.

### 3.3. Semi-Implantable Hydrogel-Based Bioelectronics

Skin-attachable and implantable hydrogel-based bioelectronics are currently making progress in monitoring electrical signals and other health monitoring applications, but both are still inherently flawed due to their location. Skin-attachable bioelectronics can only acquire limited forms of signals such as strain and electricity from the skin surface. Although information on biological macromolecules can be obtained from body fluids, the information obtained from small amounts of body fluids is still inaccurate [175]. Implantable bioelectronics can acquire signals from deep tissues. However, the biocompatibility of device components besides hydrogel cannot be guaranteed and may cause immune rejection [110].

Semi-implantable bioelectronics usually penetrate the skin stratum corneum and deep into the internal environment of the body in the form of microneedles, etc., but the main device remains on the body surface. Compared to skin-attachable and implantable bioelectronics, semi-implantable bioelectronics can obtain more accurate information and avoid harm to the body [176,177,178]. Based on the advantages of semi-implantable bioelectronics, there have been many studies in recent years using hydrogels to achieve minimally invasive health monitoring.

**Figure 11:**
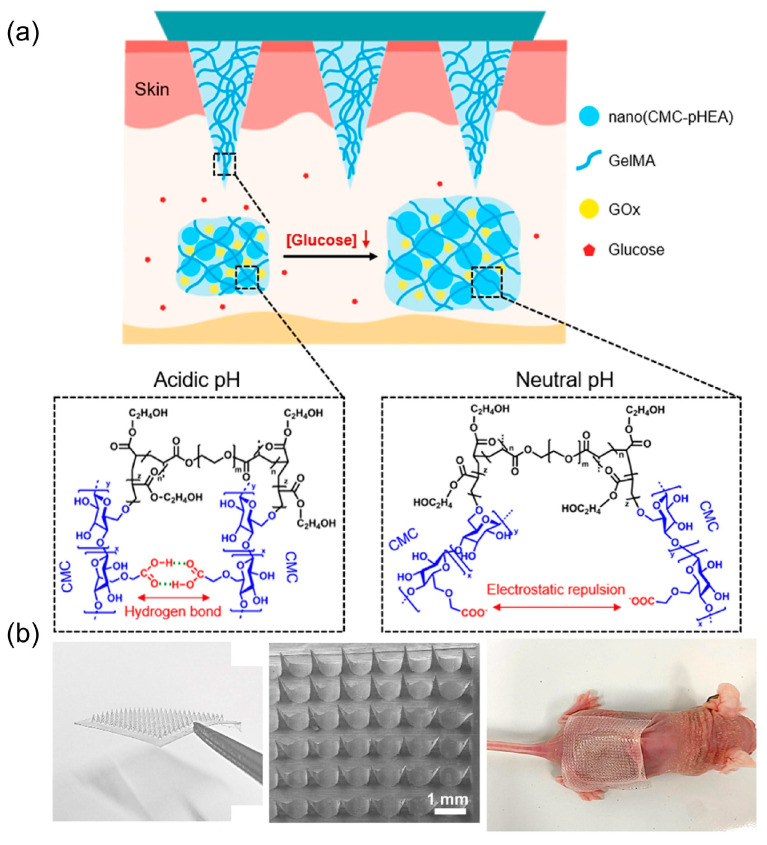
Semi-implantable hydrogel-based bioelectronics for health monitoring. (**a**) Schematic diagram and responsive mechanism of the microneedle patch system for glucose monitoring. Reprint with permission from Ref. [181]. Copyright 2022 Elsevier. (**b**) Image of cross-linked methacrylate hyaluronic acid/DNA-microneedles patch. Reprint with permission from Ref. [183]. Copyright 2022 American Chemical Society.

Hydrogels can make microneedle-absorbent body fluids for biomarker monitoring due to their water-absorbing and swellable nature [179,180]. Gelatin methacrylate (GelMA), a double-bonded modified gelatin, can be cross-linked and cured into gel by UV and visible light under the action of photoinitiators. GelMA has excellent biocompatibility, adjustable swelling, and mechanical properties and is currently a common material used in hydrogel semi-implantable bioelectronics. Wang et al. [181] achieved minimally invasive extraction and monitoring of glucose from tissue fluid based on microneedle patches, which can greatly alleviate the discomfort associated with traditional puncture to obtain blood samples for glucose monitoring (Figure 11a). The microneedles have a base diameter of 400 μm and a height of 600 μm and are made of GelMA hydrogel and pH-responsive nanogel cross-linked by light. He et al. [182] reported a “smart tattoo” based on a hyaluronic acid hydrogel microneedle patch, in which four different colorimetric reagents were loaded in the microneedle array, enabling simultaneous monitoring of pH, uric acid, glucose, and body temperature and quantitative analysis based on color changes. In addition, with the help of microneedles, semi-implantable hydrogel-based bioelectronics can obtain not only detectable macromolecules in sweat such as glucose and proteins, but also monitor microRNA in interstitial fluid [183] (Figure 11b).

## 4. Conclusions and Outlook

In this review, we summarize the basic properties of hydrogels and the application of hydrogel-based bioelectronics in health monitoring. The conductivity of hydrogels can be improved by adding CPs, conductive particles, etc. The excellent properties of hydrogels such as mechanical properties, self-healing, adhesion, and biocompatibility make hydrogel an excellent choice for health monitoring. Currently, hydrogel-based bioelectronics can be used for skin-attachable sensing, implantable sensing, and semi-implantable sensing to obtain information on human motion and biomarker content in body fluids and as flexible electrodes to collect bioelectric signals. Hydrogel-based bioelectronics can overcome the disadvantages of traditional rigid health monitoring devices to a considerable extent, greatly improving the portability and comfort of health monitoring bioelectronics. Despite the tremendous advancement that has been made on hydrogel-based bioelectronics for health monitoring, there are still some problems to be solved.

The first issue concerns the manufacturing process of hydrogels. Hydrogels have high thickness due to their own molecular structure, which does not facilitate miniaturization and multifunctional integration. Scaling down the size of hydrogel-based devices may result in decreased mechanical stability and structural integrity. Moreover, hydrogels have the property of soft and wet, which leads to the limitation of its manufacturing process and makes it difficult to manufacture high-precision structures. Water loss or swelling can lead to changes in device performance and dimensions, making precise miniaturization difficult. Therefore, new manufacturing processes need to be studied to make hydrogel-based bioelectronics more integrated and to realize multifunctional health monitoring while taking advantage of the flexibility and biocompatibility of hydrogels.

The second issue focuses on the long-term stability of hydrogel-based bioelectronics. Hydrogels have good flexibility and biocompatibility due to their extremely high water content, but hydrogel-based bioelectronics may lead to water loss and drying of the hydrogel in long-term health monitoring, which, in turn, affects the stability of the device. For example, in hydrogel electrodes for bioelectric signal monitoring, water loss can lead to unstable contact resistance and reduce the biocompatibility of electrodes. In addition to water loss, hydrogels may undergo ageing phenomena such as cross-link fracture due to chemical reactions or physical excitation. Therefore, the selection of materials used for hydrogel synthesis and the application of surface modifications are particularly important. New fillers or synthesis methods are still needed to improve the water retention of hydrogel-based bioelectronics and their stability under long-term monitoring to further expand the application scenarios of hydrogels.

The third challenge concerns the further application of hydrogel-based bioelectronics in real-time monitoring and targeted diagnosis of diseases. In terms of signal acquisition for health monitoring, the selection and specificity of biomarkers need to be considered for the application of hydrogel-based bioelectronics. Different diseases may have overlapping biomarker profiles, making it difficult to achieve high specificity. Additionally, biomarkers may exist in low concentrations, requiring sensitive detection methods and strategies to enhance signal specificity. Apart from this, the application of hydrogel-based bioelectronics requires certain ethical considerations. Ethical considerations, including privacy and data security, need to be addressed when developing and deploying hydrogel-based bioelectronics for healthcare applications.

The excellent properties of hydrogel itself make it a significant application prospect in the field of health monitoring. With the continuous improvement of its integration and stability, and the combination with the back-end data processing system, hydrogel-based bioelectronics can realize more effective real-time health monitoring and targeted diagnosis of diseases.

## Data Availability

Not applicable.
